# Development of a new rapid measurement technique for fish embryo membrane permeability studies using impedance spectroscopy

**DOI:** 10.1016/j.theriogenology.2006.02.038

**Published:** 2006-09-01

**Authors:** T. Zhang, R.Y. Wang, Q-Y. Bao, D.M. Rawson

**Affiliations:** Luton Institute of Research in the Applied Natural Sciences, University of Luton, The Spires, 2 Adelaide Street, Luton, Bedfordshire LU1 5DU, UK

**Keywords:** Zebrafish (*Danio rerio*), Embryos, Cryopreservation, Membrane permeability, Impedance spectroscopy, Cryoprotectant

## Abstract

Information on fish embryo membrane permeability is vital in their cryopreservation. Whilst conventional volumetric measurement based assessment methods have been widely used in fish embryo membrane permeability studies, they are lengthy and reduce the capacity for multi-embryo measurement during an experimental run. A new rapid ‘real-time’ measurement technique is required to determine membrane permeability during cryoprotectant treatment. In this study, zebrafish (*Danio rerio*) embryo membrane permeability to cryoprotectants was investigated using impedance spectroscopy. An embryo holding cell, capable of holding up to 10 zebrafish embryos was built incorporating the original system electrods for measuring the impedance spectra. The holding cell was tested with deionised water and a series of KCl solutions with known conductance values to confirm the performance of the modified system. Untreated intact embryos were then tested to optimise the loading capacity and sensitivity of the system. To study the impedance changes of zebrafish embryos during cryoprotectant exposure, three, six or nine embryos at 50% epiboly stage were loaded into the holding cell in egg water, which was then removed and replaced by 0.5, 1.0, 2.0 or 3 M methanol or dimethyl sulfoxide (DMSO). The impedance changes of the loaded embryos in different cryoprotectant solutions were monitored over 30 min at 22 °C, immediately following embryo exposure to cryoprotectants, at the frequency range of 10–10^6^ Hz. The impedance changes of the embryos in egg water were used as controls. Results from this study showed that the optimum embryo loading level was six embryos per cell for each experimental run. The optimum frequency was identified at 10^3.14^ or 1380 Hz which provided good sensitivity and reproducibility. Significant impedance changes were detected after embryos were exposed to different concentrations of cryoprotectants. The results agreed well with those obtained from conventional volumetric based studies.

## 1 Introduction

Although considerable progress has been made in cryopreservation of embryos of mammalian species over the last two decades, successful cryopreservation of the fish embryo has not been achieved and low membrane permeability to water and cryoprotectants has been identified as one of the major obstacles to their successful cryopreservation [1]. Conventional methods for assessing embryo membrane permeability involve the use of light microscopic measurement of volumetric change of embryos caused by exosmosis of cellular water and cryoprotectant penetration [2,3]. Other techniques have also been applied in fish embryo membrane permeability studies, including nuclear magnetic resonance (NMR) spectroscopy [4,5]. However, these methods are lengthy and reduces the capacity for multi-embryo measurement during an experimental run. Information on fish embryo membrane permeability is vital in developing successful protocols for their cryopreservation. A rapid method for assessing embryo membrane permeability during and after the treatment of cryoprotectant is needed.

Impedance (*Z*), expressed in Ω, is the ratio of the voltage impressed across a pair of terminals to the current flow between those terminals. In direct-current (DC) circuits, impedance corresponds to resistance. In alternating-current (AC) circuits, impedance is a function of resistance, inductance and capacitance. Inductors and capacitors build up voltage that opposes the flow of current. In electrochemistry, impedance spectroscopy is a well-established method for characterising the electrical properties of materials and their interfaces exposed to electronically conducting electrodes [6–9]. The technique has also been applied to the study of the dynamics of bound and mobile changes in the bulk or interfacial regions of solid and liquid materials. It has been demonstrated that impedance spectroscopy can be used as a successful tool in order to determine electrical properties of different heterogeneous systems, such as biological membrane/electrolyte systems including: conductive and capacitive properties of the bipolar membrane junction [10]; characterisation of membrane electrode assemblies in polymer electrolyte fuel cells [11] and ionic transport in a pH sensitive membrane [12]. Studies on eggs of sea urchin, rainbow trout and *Xenopus laevis* have also demonstrated the responses of their egg membranes to changes of external medium can be measured by impedance technology [13]. It could provide a novel analytical tool to study complex biological systems such as fish embryo membranes. The increasing cryoprotectant level in fish embryos should result in changes in the electrolyte that can be accurately measured by impedance spectroscopy at an optimal frequency. A rapid impedance technique for studying the dynamic behaviour of cryoprotectants and membrane permeability of the embryos would be a valuable tool in cryobiological research. In this present study, impedance value changes after embryo exposure to cryoprotectant methanol and DMSO are investigated whilst permittivity and conductivity changes after embryo exposure to cryoprotectants are reported elsewhere [14].

## 2 Materials and methods

### 2.1 Zebrafish breeding and embryo collection

Adult zebrafish were obtained from MRC Research, Hinxton, UK. Fish were held in a closed recirculating system in 12 L tanks with 8–12 fish per tank. The temperature of the system water (deionised water + 250 mg/L Tropic Marine sea salt) was maintained at 28 °C and the photoperiod was fixed at 14 h light:10 h dark. Zebrafish were fed three times daily with TetraMin flake food (Tetra, Germany) and newly hatched brine shrimps.

Embryos were collected from breeding trays each morning and maintained in egg water (60 μg/mL sea salt) at 28 °C until 50% epiboly stage. Intact embryos at 50% epiboly stage were used in this study. All embryos used for experiments were kept in egg water for at least 1 h before use.

### 2.2 Design of embryo holding well

**Fig. 1:**
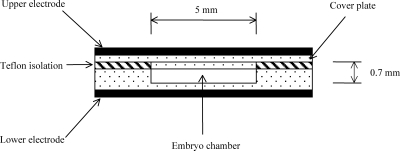
Schematic diagram of the embryo holding cell and connections with the system electrodes.

An embryo holding cell was specially designed and built for this study (Fig. 1). Both the embryo chamber plate and the cover plate were made of brass with diameters of 20 mm. The embryo chamber plate had a measurement chamber in the centre with a diameter of 5.0 mm and a depth of 0.7 mm, the remaining surface of the plate was covered by a layer of insulating Teflon. The embryo chamber was specially designed for intact zebrafish embryos which are approximately 1 mm in diameters. When embryos are loaded into the chamber, their outer membrane (chorion) should be in contact with both electrodes. Post-exposure embryo viability assessment showed that the hatching rate of the embryos was not compromised.

### 2.3 Calibration of the impedance analyser

Solartron SI1260-impedance analyzer (Novocontrol, Germany) was used in this study, the AC output voltage of the system was set at 1.0 Vrms for current measurement across the frequency range 10–10^6^ Hz with 12 logarithmic sweeping points per decade. The instrument, with the newly designed embryo holding cell, was tested with deionised water and a series of KCl solutions of known conductance values to confirm the performance of the modified system. A concentration series of KCl solutions (0.1, 0.01, 0.001 and 0.0001 M) was made up in deionised water. The concentrations of KCl were confirmed using standard bench conductivity meter (HANNA Instruments, HI 8819, UK). The embryo chamber was filled with KCl solution (approximately 0.014 mL) using a pipette and covered with the cover plate. Both plates were then secured onto the holding electrodes of the impedance analyser. The impedance values were measured with single sweeps using frequency range of 10–10^6^ Hz. The experiments were repeated six times for each concentration over 3 days. The calibrations were carried out using results from the six repeated runs for each concentration of KCl. The results were statistically analysed and quality control charts were prepared. The quality control charts were then used to confirm the performance of the instrument during the subsequent experiment period.

### 2.4 Studies of impedance changes during zebrafish embryos exposure to different cryoprotectants

Cryoprotectants methanol (METH) and dimethyl sulfoxide (DMSO) were used in the study. Methanol penetrates zebrafish embryos relatively quickly [4,15] and its toxicity to these embryos is also low [16], it was therefore chosen for this study. DMSO was also used as comparison. METH (0.5, 1.0, 2.0 and 3.0 M) and DMSO (0.5, 1.0 and 2.0 M) solutions were made up in egg water. Intact 50% epiboly stage zebrafish embryos (three, six or nine) in egg water were loaded into the embryo chamber using a pipette, the egg water was carefully removed using tissue paper, cryoprotectant solution (0.5, 1.0, 2.0 and 3.0 M METH or 0.5, 1.0 and 2.0 M DMSO) was then added to fill up the chamber. The chamber was then quickly covered and the holding cell secured between the system electrodes. Impedance values were recorded at frequency range between 10 and 10^6^ Hz with 12 frequency intervals (10^6.0^, 10^5.52^, 10^5.05^, 10^4.57^, 10^4.09^, 10^3.61^, 10^3.14^, 10^2.66^, 10^2.18^, 10^1.71^, 10^1.23^ and 10 Hz) over 30 min. The plates were polished and cleaned after each experimental run. The experiments were repeated at least nine times on different days with different spawns. Egg water was used as the control solution. All experiments were run at room temperature (22 °C).

### 2.5 Data analyses

The overall effects of applied frequency, embryo loading level and cryoprotectant concentration were statistically analysed. Student's *t*-test (two tailed assuming unequal variances) was applied to determine differences between the two contrasting groups such as the treated group and the corresponding control or two different treated groups. Values of *P* less than 0.05 were considered to be statistically significant. Means, standard errors, threshold values for *t*-test and variance were calculated using Excel.

The effect of cryoprotectant supplementation of the bathing medium (egg water) and the initial addition of embryos to the measurement cell, were allowed for in the normalisation of the impedance responses resulting from frequency changes, embryo loading levels and cryoprotectant impact.

## 3 Results

### 3.1 Measurement of baseline impedance

**Fig. 2:**
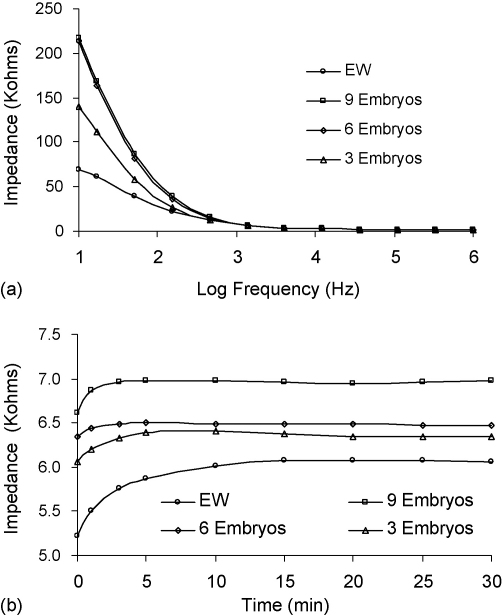
Impedances changes of embryos in egg water. (a) Impedance values measured with three, six or nine embryos loading in egg water at the frequency range of 10–10^6.0^ Hz at 30 min. (b) Impedance values measured with three, six and nine embryos loading in egg water at the fixed frequency of 10^3.14^ Hz over 30 min.

Fig. 2a shows the impedance values of different numbers (three, six or nine) of embryos in egg water at the frequency range of 10–10^6^ Hz. The results showed that at lower frequency range (10–10^2.18^ Hz) impedance values change significantly with embryo numbers, whilst at the higher frequency range (10^3.14^–10^6.0^ Hz) impedance values stayed relatively stable. Fig. 2b shows the impedance values measured from three, six or nine embryos in egg water at frequency 10^3.14^ Hz over 30 min. The results showed clear increase of impedance values with increased embryo loading levels with the highest impedance values obtained from nine embryos. No clear trends between impedance value and embryo loading level were obtained when frequencies were above 10^3.14^ Hz.

### 3.2 Effect of frequency on impedance changes during embryo exposure to cryoprotectants

**Fig. 3:**
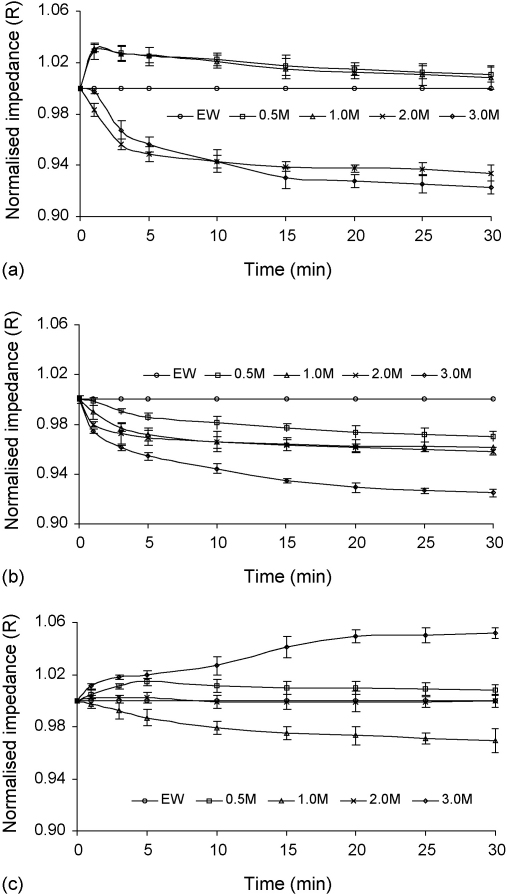
Effect of frequency on impedance change during six-embryo exposure to methanol. (a) 10^2.18^ Hz, (b) 10^3.14^ Hz and (c) 10^4.09^ Hz. The impedance values were normalised with respect to those obtained in egg water.

Impedance values measured during zebrafish embryo exposure to cryoprotectants depended on the applied frequencies. Fig. 3 shows the normalised impedance values of six embryos exposed to METH (0.5–3.0 M) at the frequency range of 10^2.18^, 10^3.14^, 10^4.09^ Hz over 30 min. The results showed that there are significant differences in impedance value changes between the embryos in egg water and the embryos exposed to different concentrations of cryoprotectants at frequency of 10^3.14^ Hz. However, the trends were not as clear for impedance values obtained at 10^2.18^ and 10^4.09^ Hz. The optimum frequency was therefore identified as 10^3.14^ Hz which provides good sensitivity and reproducibility and was used in subsequent experiments.

### 3.3 Effect of embryo loading level on impedance changes during zebrafish embryo exposure to cryoprotectants

**Fig. 4:**
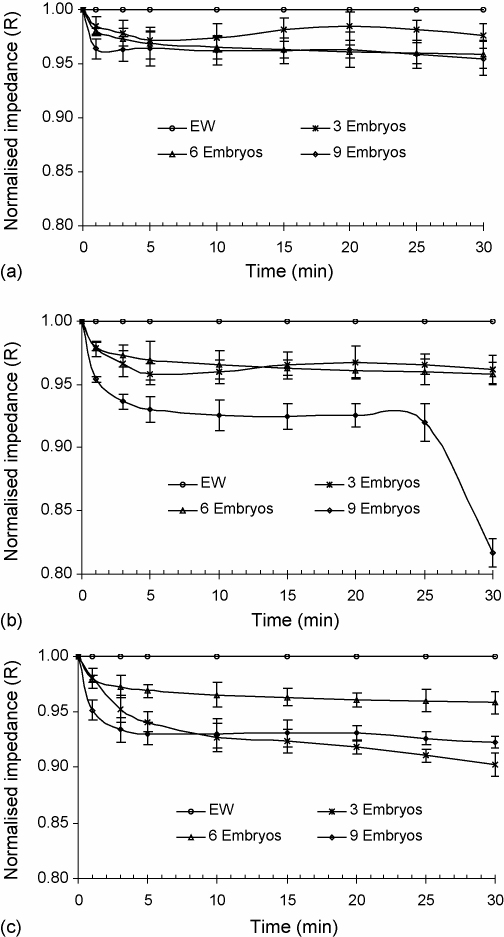
Effect of embryo loading levels on impedance change. Embryos (three, six or nine) were exposed to METH at the frequency of 10^3.14^ Hz over 30 min. (a) 0.5 M, (b) 1.0 M and (c) 2.0 M. The impedance values were normalised with respect to those obtained in egg water.

The change of impedance values not only depended on frequency used but also embryo loading level. Fig. 4 shows normalised impedance values of different embryo loading levels (three, six and nine) in METH (0.5–3.0 M) at the optimum frequency of 10^3.14^ Hz over 30 min. All three embryo loading levels in METH resulted in impedance value change. However, six-embryo loading level was the only one that resulted in steady impedance changes (decreased impedance values over exposure time) at all concentrations. Therefore, six-embryo loading level was confirmed as the optimum loading level and was used in subsequent experiments.

### 3.4 Effect of cryoprotectants on impedance change

**Fig. 5:**
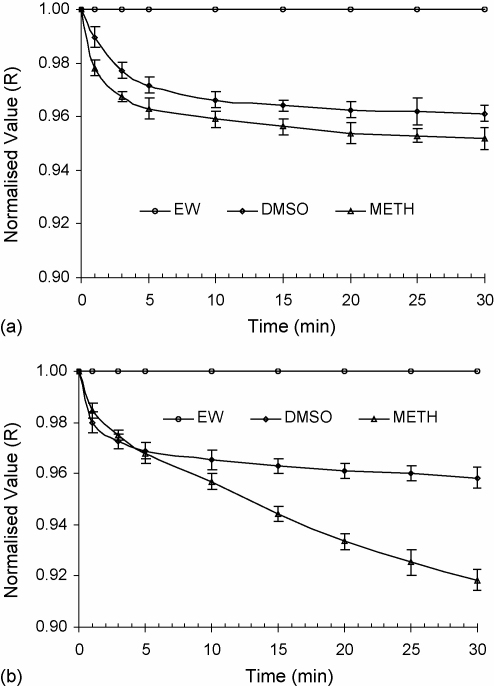
Effect of cryoprotectant on impedance change. Six embryos were exposed to METH and DMSO at frequency 10^3.14^ Hz over 30 min. (a) 1.0 M and (b) 2.0 M. The impedance values were normalised with respect to those obtained in egg water.

Fig. 5 shows impedance values of six embryos exposed to METH and DMSO (1.0 and 2.0 M) at optimum frequency of 10^3.14^ Hz over 30 min. The results showed that significant differences of impedance values were found between embryos in egg water and embryos in cryoprotectants. Comparisons of impedance changes during embryo exposure to 1.0 M METH and DMSO showed that the impedance values changed faster when embryos were exposed to METH than DMSO (Fig. 5a), indicating zebrafish embryos membrane permeability to 1.0 M METH is higher than to 1.0 M DMSO. Similar results were also found for 2.0 M concentrations (Fig. 5b). Statistical analysis also showed that the higher concentrations of cryoprotectants resulted in more significant change of impedance values. When embryos were exposed to 0.5 M METH or DMSO, no significant impedance changes were found over 30 min; for 1.0 M METH or DMSO exposure, significant impedance changes were found after 15 and 20 min, respectively; for 2.0 and 3.0 M METH exposure, significant impedance changes were detected after 3 min and for 2.0 M DMSO exposure, significant impedance changes were detected after 10 min.

## 4 Discussions

It is well documented that impedance values are frequency dependent [17,18,19]. Ferreira-Filho and Martins [20] studied isolated retina of toad and its changes during spreading depression using electrical impedance at frequency range between 10 and 50 kHz. The impedance properties of blood and packed cells have also been extensively characterised at a optimum frequency of 50 kHz [21]. Benavent et al. [22] studied the electrical behaviour of isolated tomato cuticular membranes by impedance spectroscopy measurements and found a relaxation process at frequency of around 1 and 10 kHz for both ripe and green tomatoes. In cryobiology, the impedance technique was used to determine freeze–thawing on structural changes of the membrane of biological cells in potato, sugar beet tissue and yeast suspensions [9]. The method was characterised by an accurate and rapid on-line determination of frequency-dependent electrical conductivity properties. Evaluation was based on the measurement of relative change in sample impedance at frequency between 3 kHz and 1 MHz for plant and animal cells, respectively, and 12.2 Hz and 25 MHz for yeast suspension.

Cryoprotectant penetration into embryo modifies the composition/concentration of both the external medium and intra-embryonic fluid owing to membrane osmosis and the flux of cryoprotectant, changing the ionic content and resulting in impedance/conductivity change. The design of the embryo holding cell results in the measured impedance values mainly reflecting intra-embryonic changes. The level of changes reflects the concentration of cryoprotectant solution and also depending on embryo loading level. Identify optimum loading levels of biological materials have been report to be important in impedance measurements [23,24]. So far, there are no reports in the literature on impedance measurements of fish embryo membranes during cryoprotectant exposure with which these results can be compared. The results obtained in this study demonstrate that impedance spectroscopy is clearly sensitive to cryoprotectant treatment induced impedance changes at optimum embryo loading level.

The amount of cryoprotectant penetration into the embryos depended on the concentration of cryoprotectant solutions. Embryos exposed to higher concentration of cryoprotectant resulted in higher concentration of cryoprotectant in the embryos [2,15]. This means that higher concentration of cryoprotectant exposure to embryos would result in larger impedance value change. The results obtained from the present study indicated that significant differences between untreated embryos (embryo in egg water) and embryos treated in different concentrations of cryoprotectant solutions within 30 min.

The impedance changes were qualitatively similar for both cryoprotectants METH and DMSO treatment groups but more marked for the higher concentrations. These observations were confirmed by studying impedance spectra, revealing rapid and continuing cryoprotectants permeation. Although both cryoprotectants permeated into 50% epiboly stage zebrafish embryos, METH penetration was faster than DMSO. Significant impedance changes were detected after 3 and 10 min treatment in 2.0 M METH and DMSO, respectively. These results are in general agreement with those obtained by Hagedorn et al. [5] using nuclear magnetic resonance and Zhang and Rawson [15] using real-time video microscopy. Continuous impedance measurements allowed the evaluation of the progress of cryoprotectant penetration during cryoprotectant treatment. This can provide essential data on absolute impedance value and time factors concerning the extent of cryoprotectant penetration into embryo membrane during embryo exposure to cryoprotectants. Hence, embryo membrane permeability over time can be derived from impedance value changes that were solely due to cryoprotectant penetration of the embryo membrane. One major advantage of the method lies in the reduction in time for determination of cryoprotectant penetration when compared with other traditional methods used in cryobiology. It is also a useful technique to use when cells do not maintain spherical shape during exposure to cryoprotectant solutions.

The development of a successful cryopreservation protocol requires a good knowledge of embryo membrane permeability and cryoprotectant penetration. The results obtained in this study indicated that impedance spectroscopy technique is clearly sensitive to cryoprotectant penetration induced impedance changes. These changes appeared to be frequency and embryo loading level dependent, and to vary with time during embryo exposure to cryoprotectant solutions. Under optimum conditions, significant changes of impedance values were detected during cryoprotectant treatment of the embryos, demonstrating that the technique can be used to study zebrafish embryo membrane permeability to cryoprotectants. In conclusion, the results obtained from the present study on impedance change resulting from cryoprotectant penetration into zebrafish embryos are encouraging for the continuing development of impedance spectroscopy for embryo membrane permeability studies.
